# Physical characteristics inducing Sever's disease in junior gymnasts

**DOI:** 10.1016/j.heliyon.2024.e38717

**Published:** 2024-09-28

**Authors:** Kazuaki Kinoshita, Yuichi Hoshino, Naoko Yokota, Masayuki Fukuda, Mika Hirata, Yuichiro Nishizawa

**Affiliations:** a Department of Physical Therapy, Faculty of Rehabilitation, Shijonawate Gakuen University, Osaka, 574-0011, Japan; b Department of Orthopaedic Surgery, Kobe University, Graduate School of Medicine, Hyogo, 650-0017, Japan; c Department of Rehabilitation, Kishimoto Orthopedic Rehabilitation Clinic, Osaka, 599-8121, Japan; d Center of Rehabilitation, Kobe Kaisei Hospital, Hyogo, 657-0068, Japan; e Department of Orthopaedic Surgery, Hiro sports clinic, Hyogo, 669-1535, Japan

**Keywords:** Sever's disease, Junior gymnasts, Heel, Physical, Risk factors

## Abstract

**Objective:**

This prospective cohort study aimed to determine characteristics contributing to the development of Sever's disease in junior gymnasts.

**Methods:**

This study included 74 limbs from 37 junior gymnasts. The Baseline demographic data, flexibility measurements, foot alignments, trunk function tests, and functional balancing tests were used for evaluation. The incidence of Sever's disease among this cohort was assessed for six months after the first evaluations. We categorized those diagnosed by medical specialists as having Sever's disease into a Sever's disease group, while those with no foot pain or other diagnosed pathologies were in a no-symptom group.

**Results:**

The Junior gymnasts with Sever's disease had a lower arch height ratio and lower anterior star excursion balance test values.

**Conclusions:**

Sever's disease in junior gymnasts may be potentially prevented through using a medial longitudinal arch support and/or undertaking dynamic postural balance training.

## Highlights

• Junior gymnasts with a low arch height ratio could be susceptible to Sever's disease.
• Junior gymnasts with a weak postural balancing function could be susceptible to Sever's disease.
• In this study, no difference or association was observed in ankle dorsiflexion angle.

## 1 Introduction

Sever's disease, also known as calcaneal apophysitis, is an inflammation of the growth plate in the heels of growing children, typically adolescents [1]. This disease is reported to have an incidence of 2–16 % among musculoskeletal injuries in children [2]. The pain caused by Sever's disease is often severe enough that the child limps after physical activities to take weight off the affected heel [[3], [4], [5], [6], [7]]. Most patients with Sever's disease take three to six weeks to return to painless activity [8]. Athletes with Sever's disease are often disadvantaged in being required to suspend or limit their activities during the injury period.

Sever's disease is an overuse syndrome that could be prevented by some prophylactic measures. Researchers have hypothesized potential intrinsic and extrinsic factors for Sever's disease. Mechanical stress on the calcaneal growth plate is derived from both heel contact pressure and from plantar fascia and Achilles tendon traction force and can be exacerbated by intrinsic factors such as ankle joint stiffness [9,10] and Achilles tendon tightness [11]. Extrinsic factors that have been reported to exaggerate the pathological mechanical stress on the calcaneal growth plate include high impact activities, footwear, and performing sports activities on hard surfaces [[12], [13], [14]]. Another extrinsic factor in the development of Sever's disease may involve whole-body physical function. The foot is responsible for maintaining balance through forming a supportive surface and for absorbing shock, based on kinetic chain principles. Sever's disease affects the proximal segment of the foot, which can make stabilization difficult during static and dynamic activity [15,16], with subsequent proximal segment function reciprocally affecting whole body function. For example, previous studies have shown that increased asymmetry of the multifidus is associated with non-contact lower limb injuries risk [17]. In addition, recent studies have shown that trunk muscular endurance is necessary for optimal performance and should not be underestimated [18]. Therefore, when considering undue stress on the foot, a wide range of physical functions (lower extremity muscle flexibility, joint laxity, balance, and performance) must also be taken into account.

Sever's disease occurs frequently in gymnasts. Two cohort studies have reported that gymnastics-related inversion ankle injuries are the most frequent lower extremity injuries, followed by Sever's disease [19,20]. Gymnastics involves the repetitive, jarring impact of vault takeoffs and dismounts from a variety of heights and tumbling activities. Additionally, gymnasts must perform barefoot without the support of shoes that attenuate impact during jumping and running, and, thus, Sever's disease is highly disadvantageous for gymnasts. Empirical evidence concerning risk factors or the physical characteristics in Sever's disease remains limited [21]. Therefore, this prospective cohort study, using functional tests, aimed to determine which physical characteristics of junior gymnasts might contribute to the development of Sever's disease. We hypothesized that specific risk factors related to the development of Sever's disease would be identified. The secondary objective is to explore the relationship between these identified characteristics and the incidence of Sever's disease in gymnasts. Through elucidating these relationships, the findings aim to inform preventive strategies and optimize the management of Sever's disease in this athletic population.

## 2 Materials and methods

### 2.1 Study participants

This study included 74 limbs from 37 junior gymnastics athletes, of whom 15 were males and 22 were females. The mean age, height, weight, and Rohrer index of the participants were 11.5 ± 1.7 years, 140.1 ± 10.5 cm, 33.7 ± 8.4 kg, and 120.3 ± 8.1, respectively. The participants engaged in approximately 18 h of training per week. During this period, they did not use an orthosis to prevent Sever's disease. We excluded individuals who had heel pain at the start of the study, ankle injuries and/or complaints, those with neurological or orthopedic abnormalities, individuals who complained of pain during the measurements, and those unable to complete the measurements due to the risk of further injury.

This study was approved by the ethics committee of Shijonawate Gakuen University (approval number: 20-9). The study was conducted in accordance with the ethical standards of the institutional and national research committees and with the 1964 Helsinki Declaration and its later amendments or comparable ethical standards. Informed consent was obtained from the parents or guardians and the children in this study.

### 2.2 The evaluation items

The evaluation items were 4 basic demographic data; age, height, weight and Rohrer index, 3 flexibility measurements; generalized joint laxity (GJL), heel buttock distance (HBD) and ankle dorsiflexion angle (knee flexion position), 2 foot alignments; arch height ratio and leg heel angle, 5 trunk function tests; side bridge test, abdominal muscle function, back muscle function, back and abdominal muscles strength ratio, and 3 functional balancing tests; one-legged standing, Star Excursion Balance Test (SEBT), and figure-of-8 hop (Table 1). All the items were measured by the same person. All data are expressed as mean ± standard deviation.

**Table 1:** Variables measured.

Evaluation items	Test name
Basic Demographics	Age, Height, Weight, Rohrer index
Flexibility	generalized joint laxity (GJL)
	Heel buttock distance (HBD)
	Ankle dorsiflexion angle
Foot alignment	Ratio of arch height
	Leg heel angle(LHA)
Trunk function	Side Bridge test(SB)
	Abdominal muscle function
	Back muscle function
	Back muscle/Abdominal muscle(E/F)
Functional balancing tests	One-legged standing
	Star Excursion Balance Test(SEBT)
	figure-of-8 hop

#### 2.2.1 Basic demographics

Age, height, weight, and Rohrer index were all recorded. The height was measured with a portable height scale using graduations of .1 cm. The weight was measured on an electronic scale.

#### 2.2.2 Flexibility

Generalized joint laxity (GJL) is used as an indicator of hypermobility of the joints. High values of Hypermobile athletes are considered a risk factor for ankle injuries [22]. Heel buttock distance (HBD) and ankle dorsiflexion angle are used to assess lower limb flexibility and achilles tendon tightness. These measurements have been shown to be related to the onset of Sever's disease [23].

**Fig. 1:**
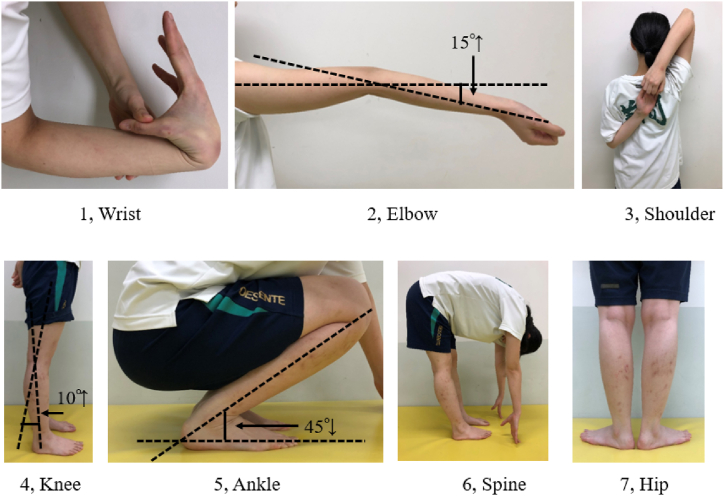
Generalized joint laxity [22,23]. Joint laxity of each participant was assessed using 7 previously described tests. This test is scored on a scale of 7 points, with each criterion earning at least 1 point. The criteria for assigning scores are outlined below. #1Wrist:The tip of the thumb touches the forearm. #2Elbow: The elbow joint extends 15° or more. #3Shoulder:Hold both hands behind the back #4Knee:The knee joint extends 10° or more. #5Ankle:The ankle joint dorsiflexes 45° or more. #6Spine:Palms of the hands touch the floor with knees fully extended. #7Hip: Straightening the knees while aligning the feet in a straight line.

GJL was measured using the University of Tokyo joint laxity test [24,25] (Fig. 1). Mobility was measured at seven positions. This test gives a total of seven points, with each item receiving one point, allowing for .5 points on each side.

#### 2.2.3 Heel-buttock distance (HBD)

**Fig. 2:**
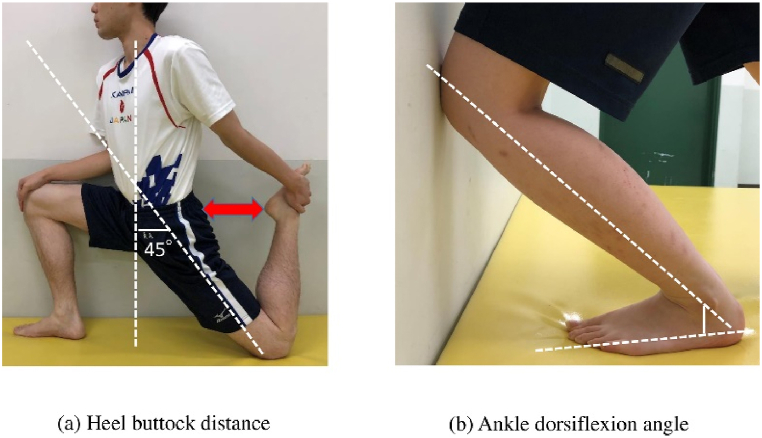
Muscle flexibility (a) In order to evaluate the flexibility of the quadriceps muscles, the knees were passively placed in flexion while the participant was in hip extension at 45° and the distance between the heels and the buttocks was measured. (b) The ankle dorsiflexion angle was measured in a weight-bearing dorsiflexion posture using a goniometer.

Generally, participants are evaluated in a prone position when assessing quadriceps flexibility [26]. This method has been employed in numerous prior studies, demonstrating a high level of reproducibility [[26], [27], [28], [29]]. however, in this study, we modified the HBD position as gymnastics athletes are very flexible. To evaluate the flexibility of the quadriceps muscles, the knees were passively placed in flexion while the participant was in hip extension at 45° and the distance between the heels and the buttocks was measured (Fig. 2a).

#### 2.2.4 Ankle dorsiflexion angle

The ankle dorsiflexion angle was measured in a weight-bearing dorsiflexion posture using a goniometer (Tokyo University) (Fig. 2b). A value of .92 has been reported to indicate excellent reliability [30].

#### 2.2.5 Foot alignment

Foot alignment is crucial for evaluating the structural balance of the foot. Arch height ratio and leg heel angle are related to the supportive function of the foot, and abnormalities in these measures can increase the risk of ankle injuries [31].

(i) Arch height rlatio

This test has shown a high Intraclass Correlation Coefficient (ICC) of .997; however, differences among participants have also been reported [32]. The foot length and the height of the navicular bone were measured using a square while the participant was in a natural standing position. The arch height ratio was calculated by dividing the height of the navicular bone by the foot length [33]. The height of the navicular bone was measured as the distance of the navicular tuberosity from the floor surface, and foot length was measured as the distance from the rear edge of the heel to the tip of the longest of the first and second toes.

(ii) Rearfoot angle [34,35]

**Fig. 3:**
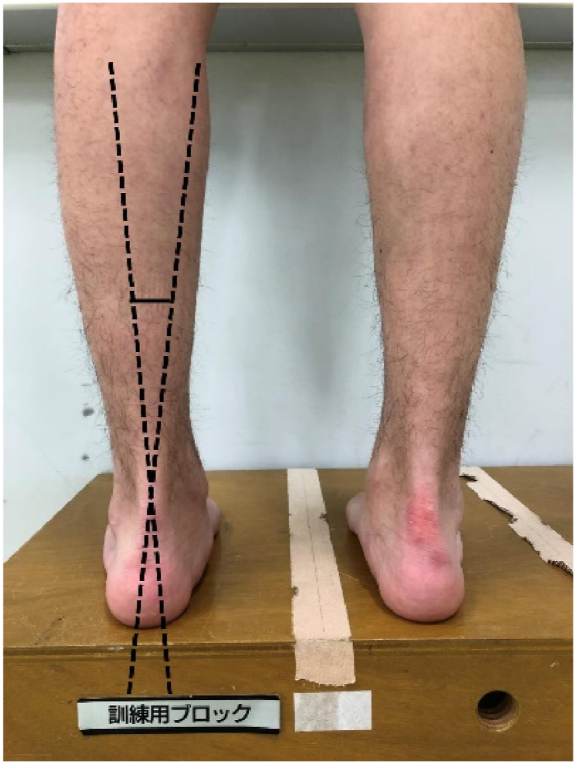
Rearfoot angle.

The angle between the long axis of the lower leg and the long axis of the calcaneus was measured, using a goniometer, with each participant placed in a neutral standing position. Dot stickers were used as references when bisecting the calcaneus and the distal leg (Fig. 3). The intra- and inter-tester reliability ICCs for this test are reported to .88 and .86, respectively [35].

The angle between the long axis of the lower leg and the long axis of the calcaneus.

#### 2.2.6 Trunk function

Trunk function plays a significant role in overall stability and athletic performance. The side bridge test, as well as assessments of abdominal and back muscle function, are widely used to evaluate trunk muscle strength and endurance. The ratio of back to abdominal muscle strength is an important indicator of trunk balance [36,37].

**Fig. 4:**
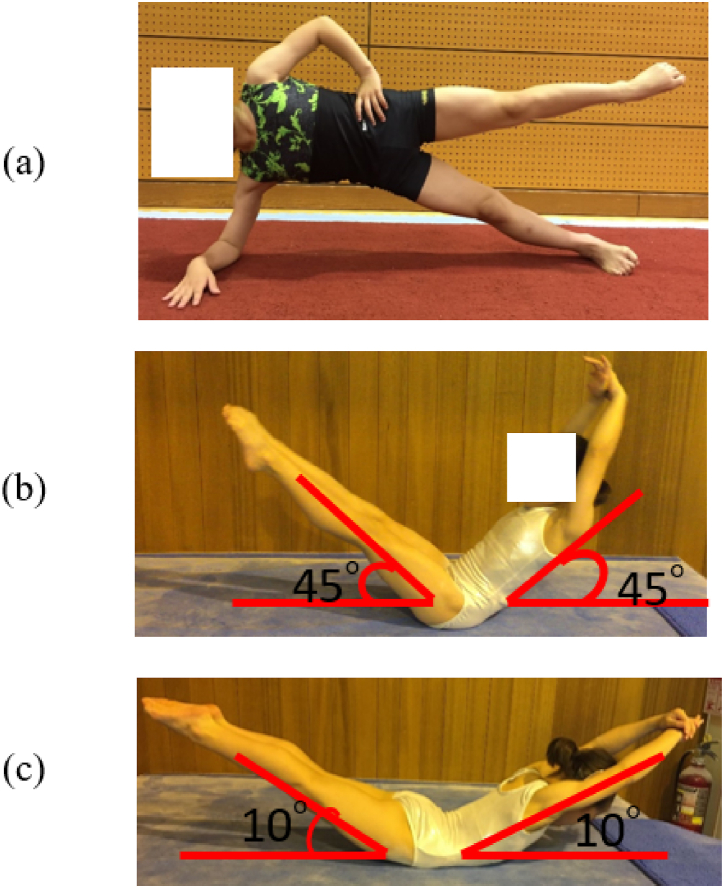
Trunk function (a) Side bridge test (b) Abdominal muscle function (c) Back muscle function.

Endurance in terms of trunk muscle function was evaluated given the importance of trunk stabilization during long practice periods. The participants were instructed to hold the position shown in Fig. 4 statically for as long as possible, and verbal cues were briefly provided to promote adherence to the form for test validity. When the participant assumed the proper position, the investigator started the stopwatch. The test was terminated when the participant failed to maintain the proper position. Tests terminated by the investigator occurred when two consecutive corrective cues given to the participants did not result in an adequate correction in form. The duration time was recorded to the nearest tenth of a second as the test results. In previous studies, the examiner was also encouraged to instruct the participants to maintain the isometric posture as long and as fully as possible for each such testing posture, and the test was performed only once. These tests have shown excellent reliability (ICC, .97–.99) [36].

(i) Side bridge test

The side bridge test was performed with the participants lying on their sides, supported by the foot and elbow. The side plank test was performed on both sides. Participants were instructed to maintain a neutral position of the spine and pelvis and to raise their lower limbs during testing (Fig. 4a).

(ii) Abdominal muscle function

The posture of abdominal muscle function is that both lower limbs and the trunk are raised 30° from the supine position, and both upper arms are attached to the ears (Fig. 4b).

(iii) Back muscle function

The posture of the back muscle function is that both the upper and lower limbs are raised 10° from the prone position (Fig. 4c).

(iv) Back and abdominal muscles' strength ratio

The back and abdominal muscles’ strength ratio was used to measure any possible imbalance between these two antagonist muscles.

#### 2.2.7 Functional balancing tests

Balance tests are used to evaluate the stability and coordination of the lower limbs. One-legged standing, SEBT, and figure-of-8 hop are standard methods for assessing static and dynamic balance [[38], [39], [40], [41], [42], [43], [44]].

(i) One-legged standing

It was measured with one-legged standing. The center of gravity sway during one-legged standing with the participant's eyes open was recorded using a stabilometer (GRAVICORDER G5500, ANIMA, Japan). To maintain their posture during measurement, the participants were instructed to fix their eyes on a target placed 2 m ahead at their eye level. Participants placed their hands on their iliac crests. Tests were conducted twice with a 1-min rest between trials. The average of two measurements was used. The center of gravity sway in one-legged standing was recorded at a sampling frequency of 100 Hz for 30 s [38]. The measurements for this test were calculated as the center of gravity sway path length per unit area (the total path length/sway area). Reliability for the static balance test has been reported as having an ICC of .90 for the dominant leg and an ICC of .91 for the non-dominant leg [39].

(ii) Star Excursion Balance Test(SEBT)

**Fig. 5:**
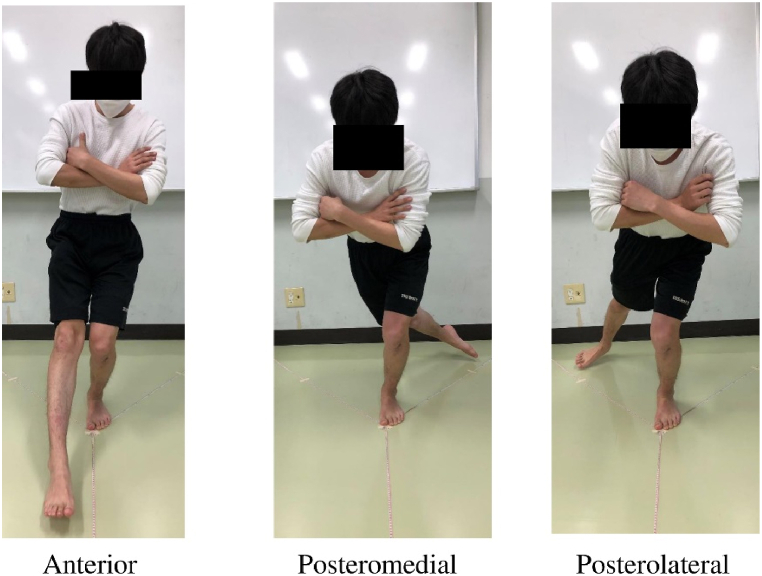
Star excursion balance test.

The SEBT is a series of single-limb squats using the nonstance limb to reach maximally to touch a point along oneof eight designated lines on the ground [40]. According to Hertel, only three reach directions (anterior, posteromedial, and posterolateral) should be performed [41], and hence, we assessed the same three directions in this study (Fig. 5). The reaching distances were normalized to the limb length of each participant. The reliability for this test has been reported to range from an ICC of .67 to an ICC of .87 [42].

The Star Excursion Balance Test is a series of single-limb squats using the nonstance limb to reach maximally to touch a point along one of eight designated lines on the ground.

(iii) Figure-of-8 hop test

**Fig. 6:**
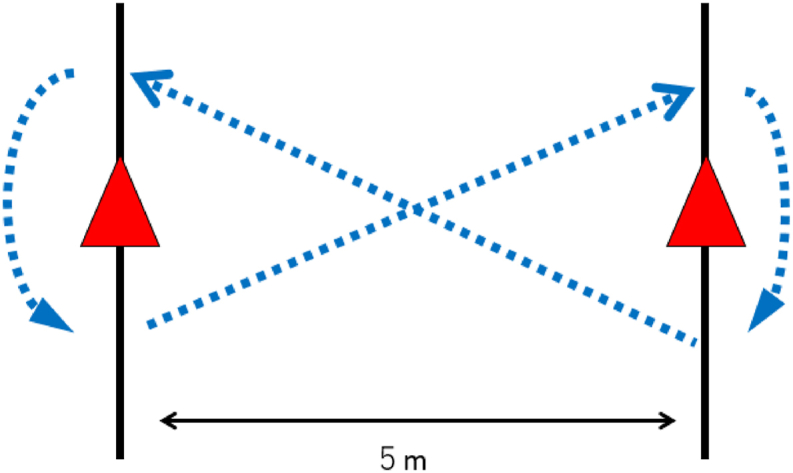
Figure-of-8 hop test.

The figure-of-8 hop test was done on a 5-m course outlined by cones. Participants were instructed to one-legged hop through the course twice as quickly as possible [43] (Fig. 6). The time to perform the test was recorded to the nearest .01 s using a handheld stopwatch. A single practice trial for patient familiarization was allowed before the test trials. The test was conducted twice on the involved limb, with a 60-s rest provided between trials. The shortest trial was used for analysis. Reliability for this test was excellent, at an ICC of .95 [44].

From a static one-legged stance, toes behind the starting line, participants perform multiple single leg hops across a distance of 5 m as quickly as possible, completing the course twice.

### 2.3 Statistical analysis

The study observed gymnasts with Sever's disease over six months from the first date of measurement. Those diagnosed at a medical institution as having Sever's disease were classified into a Sever's disease group, and those who did not have pain or who had not been diagnosed at a medical institution were classified into a no-symptom group. Participants were required to seek medical attention if they experienced pain lasting more than two weeks. If, during that examination, Sever's disease was ruled out, they were categorized into the no-symptom group. This study incorporates elements of a cohort study rather than a cross-sectional design. The Mann–Whitney *U* test was used to compare the two groups. A stepwise logistic regression analysis was used to identify factors involved in Sever's disease at six months, with the abovementioned factors as explanatory variables, and the odds ratios and 95 % confidence intervals are shown. The explanatory variables were narrowed down in advance by using the variable increase or decrease method. The input or removal values at this time were p = 0.20–.25. Statistical analysis was performed using IBM SPSS Statistics for Windows, version 20 (IBM Corp., Armonk, NY, USA). The statistical significance level was set at p < 0.05.

## 3 Results

The Sever's group comprised nine limbs (males, n = 2 limbs; females, n = 7 limbs). One male and two females exhibited symptoms bilaterally. Therefore, the total number of individuals in the Sever group was six. Measurements are shown in Table 2, Table 3, Table 4, Table 5, Table 6. The arch height ratio was significantly lower in the Sever group (12.4 ± 1.6 %) than in the no-symptom group (13.9 ± 2.1 %) (Table 4). The anterior SEBT values were significantly lower in the Sever's group (87.0 ± 5.5 %) than in the no-symptom group (94.3 ± 9.6 %) (Table 6). There were no other significant differences between the two groups. Logistic regression analysis was conducted to identify the independent relationships between Sever's disease and suspected risk factors. The explanatory variables were first narrowed down through applying a refining process. The result included three items (Rohrer index, arch height ratio, and anterior SEBT values). Finally, the arch height ratio and anterior SEBT values were applied to the model (Table 7).

**Table 2:** Measured values of the two groups of each item (Basic demographic data).

		No-symptom group (65limbs)			Sever group (9 limbs)			*p*
Age	Year	11.4	±	1.7	11.9	±	1.1	.29
Height	Cm	140.3	±	10.9	139.0	±	6.3	.72
Weight	Kg	34.0	±	8.8	31.4	±	2.7	.09
Rohrer index	kg/m^3^	120.7	±	8.1	117.3	±	7.7	.27

(Mann–Whitney U test).

∗: *p* < 0.05.

**Table 3:** Measured values of the two groups of each item(Flexibility measurements).

		No-symptom group (65limbs)			Sever group (9 limbs)			*p*
GJL	Point	3.9	±	1.6	3.5	±	.4	.23
HBD	Cm	5.7	±	6.0	5.6	±	3.3	.95
Ankle dorsiflexion angle	Angle	44.5	±	7.1	48.9	±	8.1	.09

(Mann–Whitney U test).

∗: *p* < 0.05.

**Table 4:** Measured values of the two groups of each item (Foot alignments).

		No-symptom group (65limbs)			Sever group (9 limbs)			*p*	
The ratio of arch height	%	13.9	±	2.1	12.4	±	1.6	.04	∗
LHA	Angle	7.5	±	2.3	7.3	±	1.8	.86	

(Mann–Whitney U test).

∗: *p* < 0.05.

**Table 5:** Measured values of the two groups of each item(Trunk function tests).

		No-symptom group (65limbs)			Sever group (9 limbs)			*p*
SB	Sec	64.8	±	27.1	59.6	±	39.4	.72
Abdominal muscle function	Sec	102.2	±	26.8	99.8	±	27.9	.80
Back muscle function	Sec	110.1	±	18.8	105.0	±	20.2	.46
E/F	%	95.6	±	33.6	101.4	±	41.3	.64

(Mann–Whitney U test).

∗: *p* < 0.05.

**Table 6:** Measured values of the two groups of each item(Functional balancing tests).

		No-symptom group (65limbs)			Sever group (9 limbs)			*p*
One-legged Standing	1/cm	28.1	±	8.1	27.0	±	7.2	.70
SEBT anterior	%	94.3	±	9.6	87.0	±	5.5	.03
SEBT posteromedial	%	103.4	±	11.0	100.0	±	11.0	.39
SEBT posterolateral	%	111.2	±	11.5	104.9	±	13.2	.14
Figure-of-8 hop	Sec	11.4	±	.9	11.7	±	.9	.64

(Mann–Whitney U test).

∗: *p* < 0.05.

**Table 7:** Logistic regression analysis.

	B	Standard error	Significance probability	Exp(B)	95 % confidence interval	
					Lower limit	Upper limit
SEBT Anterior	−12.967	5.418	.017	.000	.000	.095
Ratio of arch height	−.577	.249	.020	.561	.345	.915

## 4 Discussion

The main study findings indicated that there was a lower arch height ratio and reduced dynamic postural balancing function, as evaluated using the anterior SEBT, among the junior gymnasts who developed Sever's disease.

In this study, no difference or association was observed in the ankle dorsiflexion angle. Instead, the Sever's group had a greater ankle dorsiflexion angle approaching significance. Potential intrinsic factors include a limited range of ankle dorsiflexion motion [9,10]. The calcaneal growth plate and apophysis are situated in an area susceptible to high stress from the plantar fascia and Achilles tendon and may be affected by increased tension on the calcaneus [45]. Sever's disease has also been reported to be caused by tightness of the Achilles tendon [11]. But Rolf et al. questioned the existence of excessive tightness in the triceps surae. The reason is that in studies of patients with Sever's disease, multiple raters typically evaluate the dorsiflexion of the leg, which reduces the uniformity of measurement and the reliability of the results [2]. Furthermore, there are significant differences between the current study and previous literature in the methods used to assess ankle dorsiflexion angle. In previous studies examining ankle dorsiflexion angle and heel pain, participants were assessed with the knee extended [46]. Since knee extension biases the gastrocnemius muscle, while knee flexion has a Soleus bias, it is possible that tightness in the gastrocnemius muscle may have gone undetected in the case group. Therefore, some studies suggest an increase in ankle dorsiflexion due to the increased range of motion as a causative factor not previously considered [47,48]. Further discussions are required in the future.

The findings of lower arch height ratio are corroborated by prior research. Sever's disease also occurs more commonly in children who overpronate and involves both heels in more than half of the patients [49]. Sever [3] and Lewin [50] reported that there may be a slight amount of protonation present, which should be addressed. A lower arch height ratio can cause excessive stress on the surrounding soft tissues, causing muscle imbalance and abnormal joint alignment, as well as overuse syndromes [51]. Clinicians often use postural-control assessments to evaluate the risk of injury, the initial deficits resulting from injury, and the level of improvement after intervention for an injury [52].

This study yielded a new finding that the decreased SEBT values are associated with Sever's disease, which has not been previously reported. The SEBT values are used as an index of dynamic postural control (i.e., a greater distance reached indicates better dynamic postural control). High school basketball players who had a lower SEBT during the preseason were seven times more likely to sustain ankle injuries [53]. Particularly, it has a significant relationship with chronic ankle instability. Previous studies have reported that dynamic balance may better reflect the sensorimotor control mechanisms required for sports-specific tasks [54,55]. Moreover, most overuse injuries occur in the lower extremities, especially the knees, ankles, and feet. The most typical is reported to be Sever's disease [56]. Additionally, previous studies have reported that the anterior SEBT reach significantly affects ankle dorsiflexion angle and plantar sensory function compared to the posteromedial and posterolateral SEBT reaches [[57], [58], [59]]. In this study, there were no problems with the ankle dorsiflexion angle, which is considered to be related to dynamic balance. This suggests that dynamic balance is likely involved.

The overlap between these two factors may be important in the development of Sever's diseases. It has been reported that a decrease in the arch height ratio and SEBT leads to decreased lower limb support, neuromuscular control, proprioceptors, and static and dynamic balance abilities [51,52,60,61]. This may lead to overuse of the muscles around the foot and ankle joints. The arch is controlled by both local stabilizers and the global movers of the foot [62]. Local stabilizers are plantar intrinsic muscles that originate and insert on the foot; they have small moment arms and serve primarily to stabilize the arches. Many of these muscles are attached to the heel. Global movers have larger moment arms, are prime movers of the foot, and provide some stability to the arch. These typical Achilles tendons are attached to the heels. In other words, the overuse of these muscles can damage the heel. Furthermore, an unstable lower leg increases the risk of direct damage to the heel. The main theory in the literature on the pathophysiology of Sever's disease is that it is an overuse syndrome due to repetitive microtrauma caused by increased traction on the apophysis [2]. So, these two factors put strain on the calcaneus.

This study was limited owing to its small sample size. In particular, the logistic regression analysis had limited power. Additionally, the study did not separate male and female participants. Previous studies have shown significant differences in muscle stiffness and contraction characteristics between genders [63], and this is also true for gymnasts [64]. Moreover, it is possible that some participants experienced pain but did not seek medical attention, or that there was a misdiagnosis of Sever's disease, or that it was overlooked by physicians who did not consider the growth period. Some have postulated that a period of rapid growth leads to the presentation of symptoms of Sever's disease [2]. Also, practice time other than individual daily life and team practice was not considered. In addition, study participants in the no-symptom group may have experienced heel pain but did not have Sever's disease. The sampling method applied may have affected the validity of our results.

## 5 Conclusions

This study provided valuable data on the characteristics contributing to the development of Sever's disease in junior gymnasts. The main study findings indicated that there was a lower arch height ratio and reduced dynamic postural balancing function, as evaluated using the anterior SEBT, among the junior gymnasts who developed Sever's disease. In this study, there was no observed decrease in the commonly mentioned ankle dorsiflexion angle. These results will contribute to the prevention of Sever's disease. However, further research is needed to demonstrate the effectiveness of Sever's disease prevention. Future research will continue to unravel the complex interactions between Sever's disease and various factors, aiming to discover superior treatment strategies.

## Funding

This research did not receive any specific grant from funding agencies in the public, commercial, or not-for-profit sectors.

## Date availability statement

The authors do not have permission to share data.

## Ethical statement

This study was approved by the ethics committee of Shijonawate Gakuen University (approval number: 20-9). The study was conducted in accordance with the ethical standards of the institutional and national research committees and with the 1964 Helsinki Declaration and its later amendments or comparable ethical standards. Informed consent was obtained from the parents or guardians of the children included in this study.

## CRediT authorship contribution statement

**Kazuaki Kinoshita:** Writing – review & editing, Writing – original draft, Visualization, Validation, Resources, Methodology, Funding acquisition, Formal analysis, Data curation, Conceptualization. **Yuichi Hoshino:** Supervision, Project administration, Conceptualization. **Naoko Yokota:** Visualization, Supervision, Methodology, Conceptualization. **Masayuki Fukuda:** Validation, Methodology, Investigation, Conceptualization. **Mika Hirata:** Resources, Methodology, Investigation, Data curation. **Yuichiro Nishizawa:** Supervision, Conceptualization.

## Declaration of competing interest

The authors declare that they have no known competing financial interests or personal relationships that could have appeared to influence the work reported in this paper.
